# Print Quality Assessment of QR Code Elements Achieved by the Digital Thermal Transfer Process

**DOI:** 10.3390/jimaging12020086

**Published:** 2026-02-18

**Authors:** Igor Majnarić, Marija Jelkić, Marko Morić, Krunoslav Hajdek

**Affiliations:** 1 University of Zagreb, Faculty of Graphic Arts, Getaldićeva 2, 10000 Zagreb, Croatia; mjelkic@grf.hr; 2 University North, Trg dr. Žarka Dolinara 1, 48000 Koprivnica, Croatia; mmoric@unin.hr (M.M.); khajdek@unin.hr (K.H.)

**Keywords:** QR codes, UV-cured inkjet, varnish thickness, digital-thermal transfer

## Abstract

The new European Regulation (EU) 2025/40 includes provisions on modern packaging and packaging waste. It defines the use of image QR codes on packaging (items 71 and 161) and in personal documents, making line barcodes a thing of the past. The definition of a QR code is precisely specified in ISO/IEC 18004:2024. However, their implementation in printing systems is not specified and remains an important factor for their future application. Digital foil printing is a completely new hybrid printing process for applying information to highly precise applications such as QR codes, security printing, and packaging printing. The technique is characterized by a combination of two printing techniques: drop-on-demand UV inkjet followed by thermal transfer of black foil. Using a matte-coated printing substrate (Garda Matt, 300 g/m^2^), Konica Minolta KM1024 LHE Inkjet head settings, and a transfer temperature of 100 °C, the size of the square printing elements in QR codes plays a decisive role in the quality of the decoded information. The aim of this work is to investigate the possibility of realizing the basic elements of the QR code image (the profile of square elements and the success of realizing a precisely defined surface) with a variation in the thickness of the UV varnish coating (7, 14 and 21 µm), realized using the MGI JETvarnish 3DS digital machine. The most commonly used rectangular elements with a surface area of 0.01 cm^2^ were tested: 0.06 cm^2^, 0.25 cm^2^, 1 cm^2^, 4 cm^2^, and 16 cm^2^. The results showed that the imprint quality is uneven for the smallest elements (square elements with base lengths of 0.1 cm and 0.25 cm). The effect is especially visible with a minimum UV varnish application of 7 μm (1 drop). By increasing the amount of UV varnish and the application thickness to 14 μm (2 drops) and 21 μm (3 drops), respectively, a significantly more stable, even reproduction of the achromatic image is achieved. The highest technical precision was achieved with a UV varnish thickness of 21 μm.

## 1. Introduction

A Quick Response code (QR code) is a standard matrix barcode, or two-dimensional code, developed by Denso Wave in 1994 for Toyota’s production line. A matrix barcode is a pattern of black and white squares used to store data. Its main advantage is the ability to store substantial data in an image that can be quickly decoded and read. The code consists of square black modules (individual squares) on a white background. The arrangement of modules forms unique binary (ones and zeros), alphanumeric (letters and numbers), or Kanji (Japanese characters) coded information [1,2].

Although global product identification still uses the fast, accurate, and fifty-year-old two-dimensional linear bar code (present on more than a billion products and scanned billions of times every day), marking products with the QR code system has become popular in the consumer goods labeling sector (scanning the code brings the customer to the manufacturer’s website, where more detailed text and image information, as well as video content, can be obtained) [3,4,5].

It is expected that by 2027, the QR code will replace the traditional barcode, enabling the necessary transformation to meet the needs of traceability, safety, and sustainability. This also provides detailed information on the product’s nutritional properties, the composition of its packaging and textiles, and links to websites where additional information can be shared [6,7].

As the needs of consumers and producers become increasingly complex, new EU directives and legal regulations are being applied to the use of QR codes. It started with the directive to introduce QR codes on European driver’s licenses and with current regulations on packaging and packaging waste [8,9].

The symbology and application procedure of QR codes are defined in detail in ISO standards per ISO/IEC 18004:2015 and ISO/IEC 18004:2024 [10]. Their differences are reflected in two amendments: one related to continuous grading (per ISO/IEC 15415:2024, adopted for grade-fixed pattern damage), and the other clarifying the reference decoding algorithm [11]. Normative references directly implemented in these standards are prepared by Joint Technical Committee 1 Subcommittees SC 31 (ISO/IEC 8859-5:1999; ISO/IEC 646:1991; ISO/IEC 6429:1992; and ISO/IEC 8859-1:1998) [12,13,14,15].

## 2. Theoretical Part

### 2.1. Structure of QR Symbols

Depending on the size of the data to be measured, we can distinguish between two basic families of standard codes: QR codes and micro QR codes. Symbolic QR codes are available in 40 sizes (from Version 1 to Version 40), while micro QR codes are smaller and available in 4 versions (M1, M2, M3, and M4). For such codes to work, each symbol must be built from nominally square modules arranged in a regular square array, with the quiet zone surrounding it on all four sides. QR codes must necessarily contain two areas: the encoding region and the functional pattern area. Functional patterns do not encode data and contain: (I.) a quiet zone, (II.) a finder pattern, (III.) a separator, (IV.) timing patterns, and (V.) alignment patterns [15].

**Figure 1:**
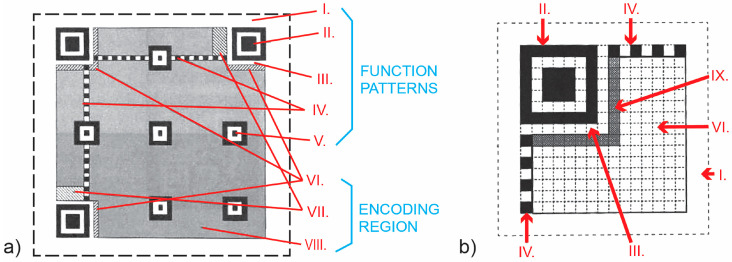
Symbol structure: (**a**) standard QR code version 7; (**b**) micro QR code version M3 [10].

**Figure 2:**
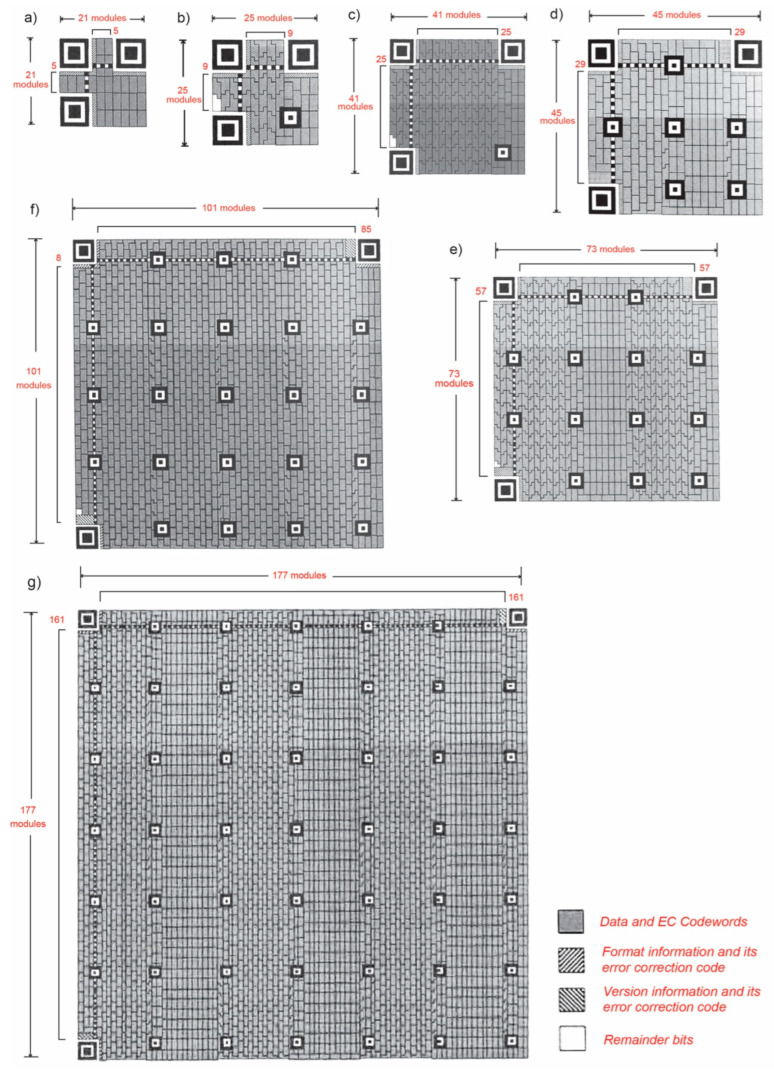
Type of QR symbol module: (**a**) version 1, (**b**) version 2, (**c**) version 6, (**d**) version 7, (**e**) version 14, (**f**) version 21, and (**g**) version 40 [10].

**Figure 3:**
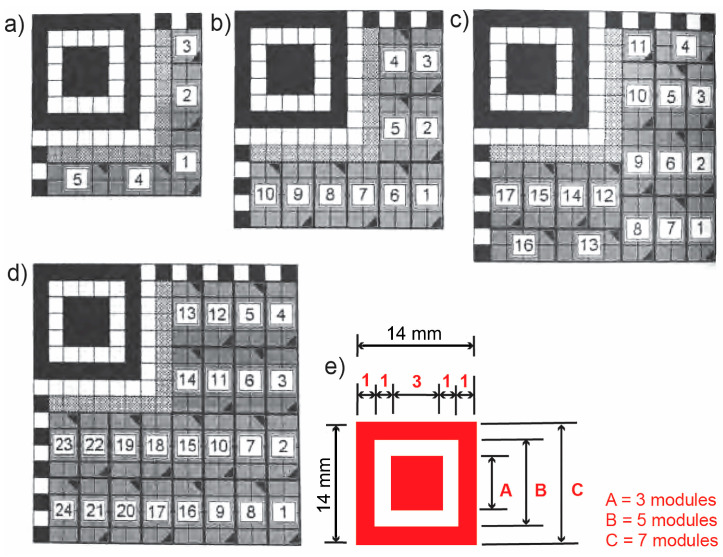
Dimensions of the micro QR symbol module: (**a**) version M1, (**b**) version M2, (**c**) version M3, (**d**) version M4, (**e**) definition of the finder pattern [10].

There are always three identical finder patterns in a QR code, located in the upper-left, upper-right, and lower-left corners of the symbol (Figure 1a and Figure 2). Each finder pattern can be viewed as three precisely positioned concentric squares, each consisting of 7 × 7 dark modules, 5 × 5 light modules, and 3 × 3 dark modules. The module width ratio in each finder pattern is 1:1:3:1:1 (Figure 3e). The symbol is preferentially encoded so that similar patterns are unlikely to occur elsewhere within the symbol. This enables it to be rotated and inverted, facilitating rapid identification of a possible QR code in the field of view.

Alignment patterns consist of three precisely positioned concentric squares of 5 × 5 dark modules, 3 × 3 light modules, and one central dark module. The number of generated alignment patterns depends on the QR version; it is present in QR symbols starting with version 2. Imaging patterns always begin and end with a dark module. By definition, they consist of a row or column one module wide with alternating dark and light modules [16].

Encoding regions are areas for additional information, such as format information (VI.), version information (VII.), and data and error-correction codewords (VIII.). Four levels of Reed–Solomon error correction can be used when interpreting codes. These are: L (7% data recovery), M (15% data recovery), Q (25% data recovery), and H (30% data recovery). This ensures the readability of data and symbol codewords even if the elements are deformed during printing.

The micro QR code symbol must also be surrounded by a quiet zone. However, instead of three finder patterns, the micro QR code symbol contains only one finder pattern (Figure 1b). The functional patterns are unchanged and contain: (I.) a quiet zone, (II.) one finder pattern, (III.) a separator, (IV.) edge timing patterns, and (V.) format information. This means that in the encoding region, (IX.) there are no alignment patterns, while the finder pattern is located in the upper left corner and is defined identically to other QR codes (Figure 3e).

The digital process of converting input data into a QR code symbol is performed in seven steps. The process begins with data analysis and encoding into 8-bit code words. This is followed by encoding error correction words and structuring the final message. This is followed by matrixing the modules (adding separators, search patterns, time patterns, and alignment patterns) and data masking (optimizing the balance between dark and light modules). Finally, information about the format and the produced version is generated [17].

#### 2.1.1. QR Codes

The simplest standard QR code (version 1) measures 21 modules × 21 modules whose function pattern modules number 202, which ultimately enables a data capacity of 44 codewords. Informationally, the largest QR code (version 40) is a matrix module of dimensions 177 × 177, with a function pattern of size 1614, which enables a data capacity of 3706 codewords. Visually, this allows for the display of: 7089 numeric characters, 4296 alphanumeric characters, 2953 byte characters and 1817 Kanji characters [8]. 

In the standard versions of the QR code (from version 1 to version 40) changing the size will achieve an increase to the size in steps of four modules). Figure 2 shows the definitions of seven characteristic QR codes [18,19].

#### 2.1.2. Micro QR Codes

Due to the dimensions of micro QR codes, they enable lower record capacities. Thus, the M1 version will measure an 11 × 11 module with a functional pattern of 70 and a capacity of five codewords, while the largest micro QR code will have dimensions of a 17 × 17 module, a functional pattern of 82 modules, and a capacity of 24 codewords. Visually, the M4 micro QR code allows for the display of: 35 numeric characters, 21 alphanumeric characters, 15 byte characters, and 9 Kanji characters. In this type of code, the version changes and the size increases sideways in steps of two modules. Figure 3 shows the four types of micro QR codes and the pattern for finding them.

**Figure 4:**
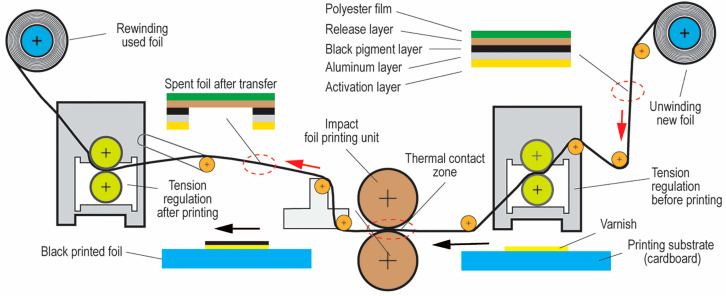
Schematic drawing of the printing and foil transfer process.

Digital and analog printing technologies can directly affect the reproduction quality of the smallest elements of the formed module. In this case, the printing unit (pressure, printing plate, and printing method), the type of ink (viscosity and ink layer thickness), and the type of printing substrate (whiteness, absorbency, structure, and smoothness) all play an important role [20]. Printing with additional thermal transfer foil is led by the companies Leonhard Kurz Stiftung, Scodix, MGI, and Duplo. Their systems are used to print on paper and cardboard products in various print dimensions and constructions. A common feature among these machines is the use of specially designed foils, with layer compositions varying according to thickness, color, and adhesive properties [21,22,23]. Figure 4 illustrates the principle of printing with the Digital Thermal Transfer Process, which uses Inkjet technology to apply UV varnish on printing substrate [24]. 

Numerous research papers by the author concern printing systems and the reproduction of the basic linear element as the basis of coding. However, now the focus is the rectangle inside the QR code module. The optimal size of the QR code module depends on the purpose and scanning method. For mobile device scanning at close range, a module size (square) of 0.4 to 0.5 mm is recommended, while for scanning from a longer distance, the module size can be increased to 0.5 to 1 mm. For reliable reading, industrial or automatic readers require modules with a size of 0.7 to 1.2 mm. The minimum module size should be about 0.36 mm when the QR code is printed on small packages or labels. For larger printed media and packaging, modules should be larger than 1 mm, so the code can be easily read from a greater distance. Printing techniques (printing speed, machine design, applied printing form, and inks), as well as the printing substrate, play a crucial role [25].

The smallest size of a square QR code element (module) that is technically achievable with UV varnish or the digital thermal transfer process is approximately 0.36 × 0.36 mm [25]. Below this value, there is a risk of droplets sticking together, insufficient edge definition, and reduced readability. The frequent module size for UV application of QR codes is 0.6–0.8 mm. This size provides sufficient space for the precise design of each element, preservation of contrast, and reliable machine reading, even with minor substrate curvature or minor optical damage (even with partial reflections) [26]. In addition, factors such as relief height, contrast relative to the substrate (especially with transparent varnish), error correction level (level M or H is recommended for touch-up techniques), and the possibility of rotational scanning should be considered. Also, QR codes printed with UV varnish or digital thermal transfer foil should be tested with standardized readers. ISO/IEC 15415:2024 is used to verify their functionality in real-world application conditions [11].

The digital printing of QR codes using inkjet UV varnish machines with subsequent thermal transfer foil is an advanced technique for integrating data into printed products, with additional esthetic and security functions.

Successful implementation requires the precise creation of a bitmap mask, knowledge of minimum dimensional requirements, and validation of the readability of the finished codes. Properly designed and applied QR codes using this method enable high levels of personalization, tracking, and user interaction across a variety of contexts, from luxury packaging to digital identification [27,28,29,30].

#### 2.1.3. Reading QR Codes

Reading QR codes displayed on a screen is much easier than reading printed QR codes. The readability of QR codes depends on the resolution and contrast of the image. Depending on the characteristics of the original image, it can sometimes be easier to read the QR code from a screen than from a printout. Scanning and reading QR codes today is done with various devices, the most common of which are smartphones and tablets, which use high-resolution cameras and built-in applications to read codes.

Handheld scanners are mainly used in the business and logistics sectors. These are portable devices that use laser or image technology to read codes and connect to computers via a USB port or wirelessly. A more advanced system uses handheld terminals that, in addition to sensors, have an integrated operating system and enable data processing, storage, and transmission. They are particularly useful in warehouse management, logistics, and industry. Fixed scanners are installed in fixed positions (e.g., cash registers, production lines, or facility entrances), enabling fast, precise decryption of coded content [31].

There are several manufacturers of 2D code scanners on the market to measure parameters such as readability, contrast, sharpness, positioning, and print quality. These scanners play a crucial role in ensuring reliable identification and traceability across industries such as retail, logistics, production, and quality control. Among the most famous companies are: Zebra Technologies with its DS2200 and DS8100 series (USA), Honeywell Xenon series and Granit series (USA), Datalogic Gryphon series and Magellan series (ITA), Cognex DataMan series (USA), Keyence (JAP), and REA Elektronik with its REA Verifier, REA VeriCube IR and VeriCube UV (GER) systems [32,33,34,35,36].

In this paper, the aim is to analyze the feasibility of implementing the basic structure of a QR code using a new hybrid digital thermal foil printing process. In papers [37,38], different methods of applying UV-curable inkjet varnish—which refers to a liquid coating cured by ultraviolet light—have been investigated, but not the subsequent thermal transfer of the colored foil, a process in which heat is used to bond the colored foil to a surface [39,40,41,42,43,44].

Printed black foil on white cardboard generates an extremely high optical density (D > 3.0), meaning the printed areas are very dark and non-reflective, which should provide satisfactory contrast. However, edge bleeding of the UV inkjet varnish (glue) can create problems in realizing the smallest square elements (micro QR codes), i.e., native low-resolution piezo inkjet heads apply a much larger amount of UV varnish (droplets) than necessary [45].

## 3. Materials and Methods

For the experiment, an achromatic digital printing form was generated in Adobe Illustrator 2024. In order to simulate the reproduction of potential QR codes, the digital printing form contains a synchronization strip, standard QR codes of different sizes and square elements of different surfaces, i.e., page sizes of 0.01 cm^2^ (0.1 cm); 0.06 cm^2^ (0.25 cm); 0.25 cm^2^ (0.5 cm); 1 cm^2^ (1 cm); 4 cm^2^ (2 cm) and 16 cm^2^ (4 cm).

For printing, the SRA3 digital printing form was converted to bitmap files using Adobe Photoshop 2024. This is necessary so it can be used in the JETvarnish 3DS machine as a mask for the AIS scanner (to precisely position the UV varnish on the print). The generated digital record is exported as a PDF file, which also constitutes a reference sample (S1). Such a file format is suitable for processing in RIP MGI Juti 3.2, with which the desired application of UV varnish can be applied in a controlled manner [46].

Experimental printing was performed on the MGI JETvarnish 3DS printer (all print runs are 30 pcs in 3 conditions), which uses five KM 1024 iLhe-30 inkjet heads [47]. During the experiment, three thicknesses of UV varnish (JV3DS-LED Varnish) were applied: 7 μm (1 drop), 14 μm (2 drops), and 21 μm (3 drops). A transfer temperature of 100 °C was applied for the final bonding of the UV varnish and black MGI iFoil foil. This is also the manufacturer’s recommended setup temperature. At this temperature, potential regulation of ±30 °C is possible. That means that potential regulation can be in the range of 70 to 130 °C. The experiment was executed under constant climatic conditions (temperature at 23 ± 2 °C and humidity at 50 ± 5% RH). Commercially available GardaMatt cardboard (300 g/m^2^) was used as the substrate. Based on experimental variations in the inkjet head settings, three types of patterns were applied to matte-coated cardboard, containing square elements with base sizes of 0.1 cm, 0.25 cm, 0.5 cm, 1 cm, 2 cm, and 4 cm. All experimentally produced patterns were scanned on an EPSON L3266 scanner at a resolution of 600 dpi, and the images were processed in Adobe Photoshop (final dimensions 49 × 49 mm^2^, saved as a bitmapped image in JPG format). The prepared images were then analyzed with the ImageJ 1.54 g software, using the following macro code:

run(“Set Scale…”, “distance = 1157 known=49 unit = mm global”);

run(“Invert”);

run(“Set Measurements…”, “area mean min perimeter area_fraction

redirect = None decimal = 3”);

run(“Measure”);

This code thus defines the steps applied when authors perform image analysis. For precise testing, profiles were generated only for characteristic elements with base lengths of 0.5 cm, 1 cm, and 2 cm. These are also the most common methods for reproducing QR codes in the graphics industry. Finally, profiles were generated, and the printed surfaces of characteristic square elements were analyzed. In this process, an achromatic image with 256 shades of black was reduced to an image in the saturation range from 0 to 100% using Equation (1):

*D* = *y*/256 × 100% (1)

where D = darkness value of the printed element, and y = measured grayscale of the printed image in greyscale mode.

**Figure 5:**
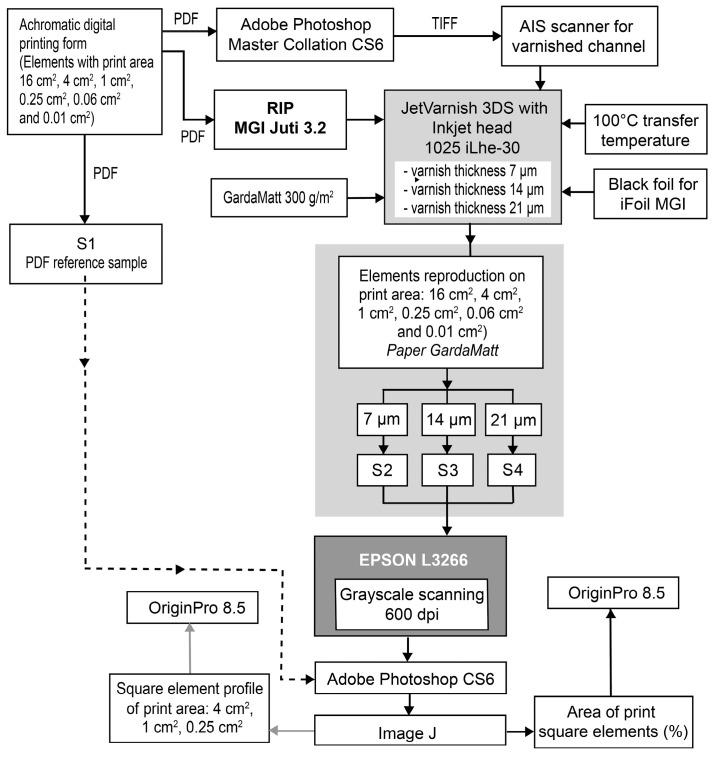
Scheme of the performed experiment.

When printing square elements (with equal height and width), the substrate and inkjet head speeds differ. This difference affects the final print because the machine printing direction (MD) and cross direction (CD) are misaligned. Therefore, this paper focuses on the MD, while the CD will be explored in future work. The results graph was created using OriginPro 8.5. The chronological flow of the performed experiment is shown in Figure 5.

## 4. Results and Discussion

**Figure 6:**
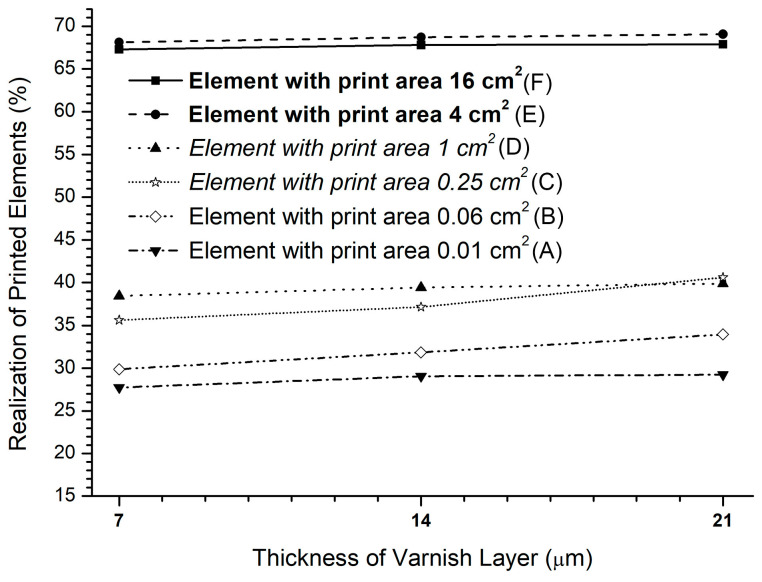
Realizing six different-sized print elements.

**Figure 7:**
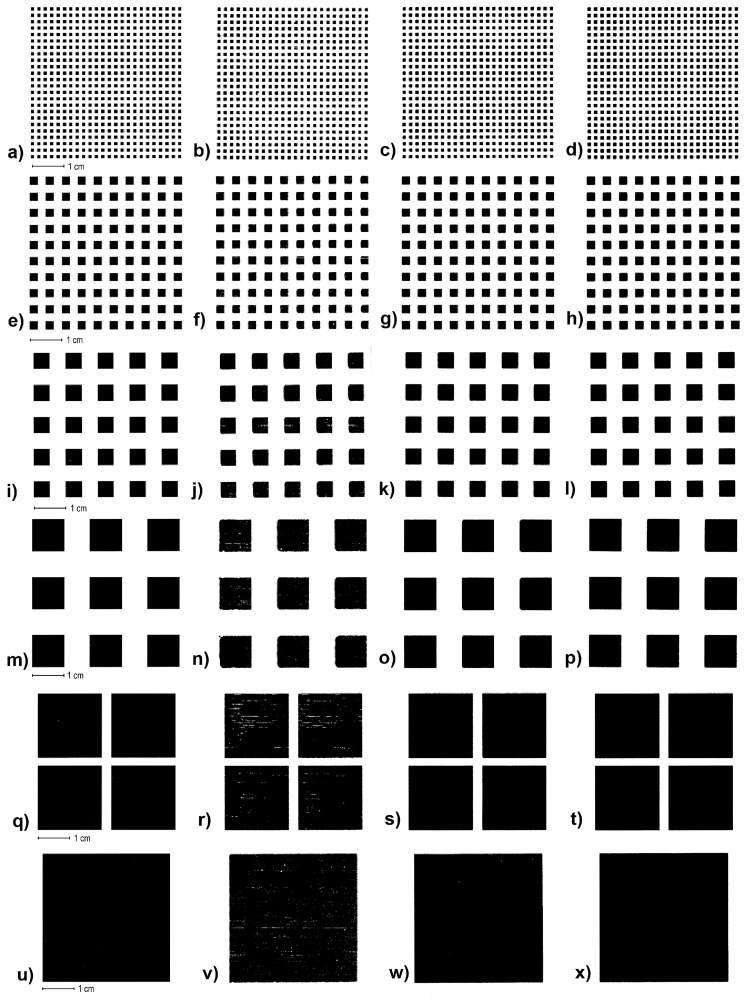
Images of scanned experimentally printed samples with a square element: (**a**–**d**) 0.01 cm^2^ (reference, 1 drop, 2 drops, 3 drops,); (**e**–**h**) 0.06 cm^2^ (reference, 1 drop, 2 drops, 3 drops); (**i**–**l**) 0.25 cm^2^ (reference, 1 drop, 2 drops, 3 drops); (**m**–**p**) 1 cm^2^ (reference, 1 drop, 2 drops, 3 drops); (**q**–**t**) 4 cm^2^ (reference, 1 drop, 2 drops, 3 drops,); (**u**–**x**) 16 cm^2^ (reference, 1 drop, 2 drops, 3 drops).

The experimentally printed square elements were divided into three-dimensional groups: two large elements (**area is 16 cm**^2^ **and 4 cm**^2^), two medium-sized elements (area is *1 cm*^2^ *and 0.25 cm*^2^), and two small elements (area is 0.06 cm^2^ and 0.01 cm^2^). The results in Table 1 and Figure 6 show achieving a surface coverage (A% = area percentage of printed black foil) for the selected print elements within the region of interest (ROI) of 24.01 cm^2^ (4.9 × 4.9 cm). Figure 7 shows enlarged images of the realized prints and graphs of the percentage of the realized six differently sized square elements with UV varnish coatings of 7, 14, and 21 μm on matte fine art cardboard.

**Figure 8:**
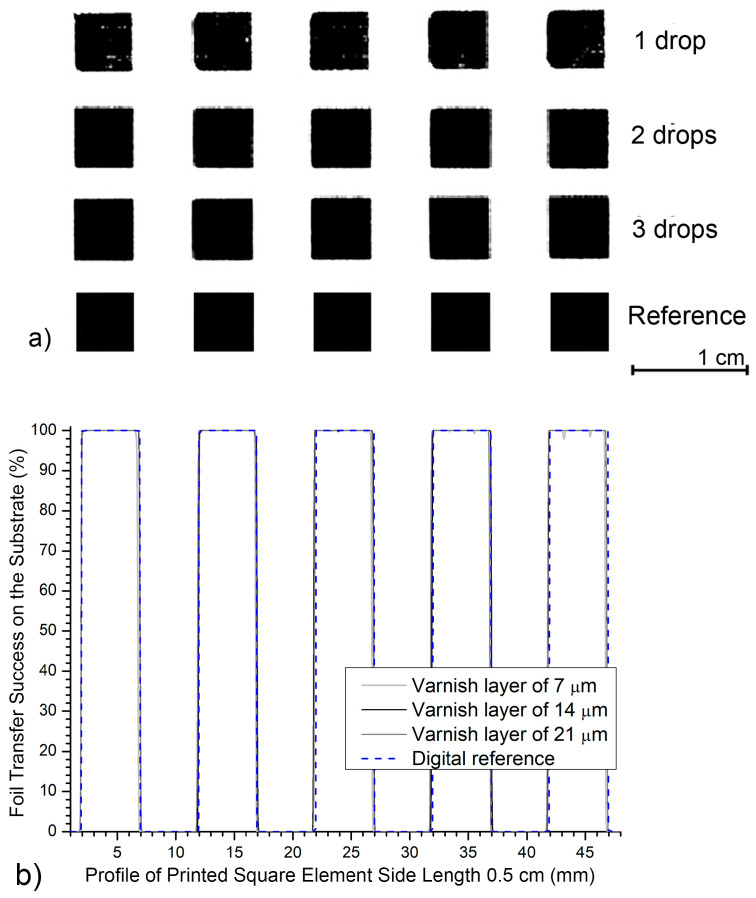
Five printed elements created on a matte-coated substrate on a surface 5 cm long: (**a**) photo of a sample magnified 2×; (**b**) generated profiles.

Figure 8 shows the data of the six sizes of square elements realized on matte-coated cardboard. A very slight and stable increase in the realization of all printed elements was noticed with an increase in the thickness of the varnish coating. At coatings of 7 and 14 μm, it is visible that the “smallest” printing element (area of 0.01 cm^2^) significantly deviates from the value of the realization of the same printing element realized at the highest varnish coating (21 μm). The smallest printed element, thus, at a varnish coating of 7 μm is *A*_S2_A_ = 27.7%, at a varnish coating of 14 μm it is *A*_S3_A_ = 29.0%, while at a varnish coating of 21 μm it is *A*_S4_A_ = 29.2%.

The second printing element in size (square area of 0.06 cm^2^) has similar final results. With a varnish application of 7 μm, the realization is *A*_S2_B_ = 29.8%; with a varnish application of 14 μm, the realization is *A*_S3_B_ = 31.8%; while with a varnish application of 21 μm, the realization is *A*_S4_B_ = 33.94%. Comparing it with the results of the implementation with larger UV varnish applications, the differences are Δ*A*_S2_S3_ = 2.1% and Δ*A*_S1_S3_ = 4.1%.

The group with medium-sized printing elements has the lowest realization values when applying a 7 μm varnish. They are *A*_S2_C_ = 35.6% for a printing element with a square area of 0.25 cm^2^ and *A*_S2_D_ = 38.4% for a printing element with a square area of 1 cm^2^. When comparing these two elements, a difference of Δ*A*_S2C_S2D_ = 10.7% is observed. A single increase in varnish application results in a small increase in the printing element realization. They reach their maximum when applying a varnish of 21 μm, which numerically amounts to *A*_S3_C_ = 37.14% and *A*_S4_D_ = 40.62%.

In relation to the smallest element, the differences are: Δ*A*_S2A_S2C_ = 7.9%; *A*_S3A_S3C_ = 8.0%; and *A*_S4A_S4C_ = 11.4% (which is also the largest deviation for that element). The differences in the realization of an element with a square area of 0.06 cm^2^ and 0.01 cm^2^ are smaller and amount to Δ*A*_S2B_S2A_ = 2.2%, Δ*A*_S3B_S3A_ = 2.8% and Δ*A*_S4B_S4A_ = 4.8%.

With increasing varnish layer thickness, “large” elements can be better realized, leading to minimum values of realization (*A*_S2_E_ = 68.1% and *A*_S2_E_ = 67.2%). The highest rates in realizing black printed surfaces are achieved with the largest varnish layer (21 μm), which yield *A*_S4_E_ = 69.0% and *A*_S4_F_ = 67.8%. The graph shows that the element with a 4 cm^2^ area has the highest realization value. This value is greater than for the element with a 16 cm^2^ area (Δ*A*_S4E_S4F_ = 1.2%).

Therefore, the largest printing elements show the stability of the implementation at all thicknesses of the UV varnish coating, which is also shown by the deviation values of Δ*A*_S2F_S4F_ = 0.6% (for an element with an area of 16 cm^2^) and Δ*A*_S2E_S4E_ = 1.0% (for an element with an area of 4 cm^2^). It is visible that the achieved values of these deviations are significantly smaller compared to the deviations of all other square printing elements.

Figure 8 shows the profile of five printed square print elements with a base side length of 0.5 cm, applied with UV varnish on a matte-coated printing substrate. Compared with the reference profile of an ideal square shape, results are presented for three different varnish layers (7, 14 and 21 µm) over a 5 cm length. This analysis shows how different UV varnish layers behave on a matte-coated substrate when transferring smaller graphic elements. For all three UV varnish thicknesses (7, 14, and 21 μm), the surface profiles show precise transfer of the square pattern.

With a 7 μm UV varnish layer, the dark values remain high and stable (highest measured value *D*_S2_Cmax_ = 100.0%, lowest *D*_S2_Cmin_ = 97.9%). Thus, the total amplitude of the darkness deviations from the target value (i.e., the variation in measured darkness levels across the area) is Δ*D*_min_max_ = 2.1%. The surface of the printed element is uniform, and deviations from the target value indicate good acceptability of the black foil on matte cardboard, even with the smallest layer achieved.

A 14 μm (medium layer) of UV varnish provides an almost ideal profile. The edges are completely aligned with the reference, and the surface is stable and completely flat. Dark values range from *D*_S3_Cmin_ = 99.8% to *D* S_3_Cmax_ = 100.0%, with negligible darkness deviations from the target value of Δ*D*_min_max_ = 0.2. This variant provides the best balance between the amount of varnish and control of the black print, confirming 14 μm as the optimal value even for smaller printing element dimensions. At the largest experimental deposit of 21 μm, the surface remains equally flat (without visible deformations).

**Figure 9:**
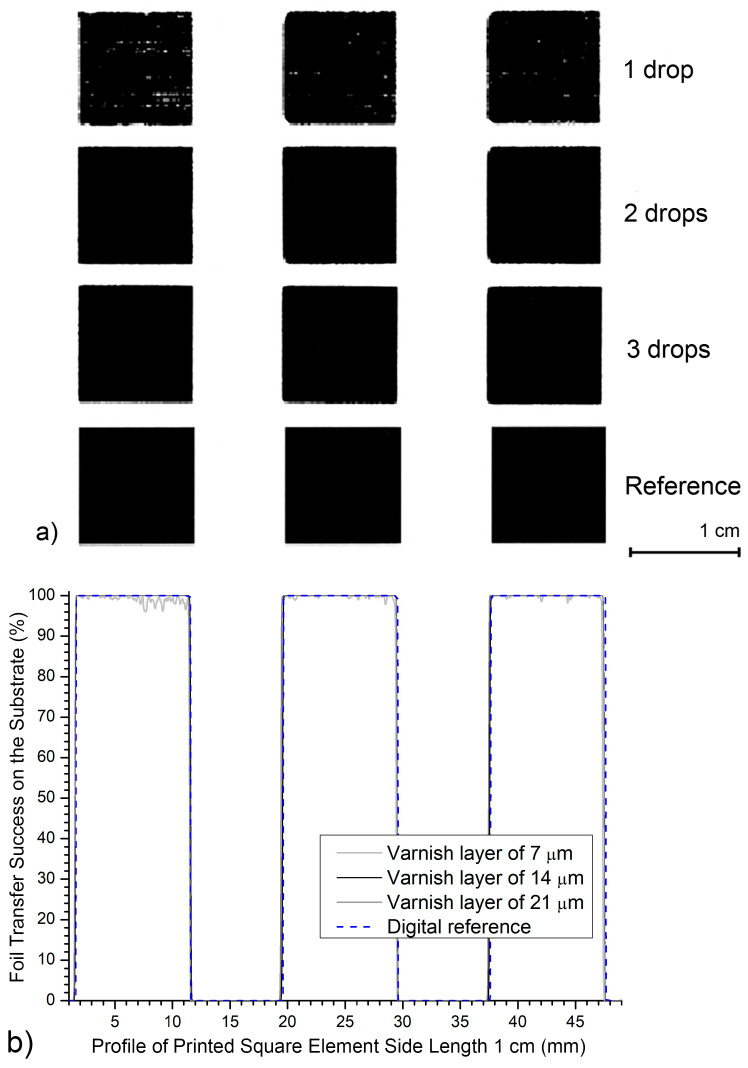
Three print elements created on a matte-coated substrate on a surface 5 cm long: (**a**) photo of a sample magnified 2×; (**b**) generated profiles.

Figure 9 shows the profile of three square printing elements with an area of 1 cm^2^. The profile was created by measuring the squares printed with UV varnish on a matte substrate and selecting a 5 cm long image. The graph includes three varnish layers (7, 14 and 21 μm) and a reference square profile of an ideal impression.

For the smallest 7 μm layer, a slight increase in fluctuations is observed within the black printed element. The surface values sometimes dip below *D*_S2_D_ = 100.0%. The edges remain well-defined and match the reference profile, but the graph shows minor irregularities within the square structure. The printed surface ranges from 100.0% to 96.0%. This means the surface oscillates by Δ*D*_min_max_ = 4.0%.

The 14 μm UV varnish layer shows an almost perfectly matched profile to the reference. The surface inside each black square is flat *D*_S3_D1_ = 100.0%. The edges are clearly defined. Deviations from the target value are minimal, appearing only at the edges of the black printing elements. At the maximum UV varnish layer (21 μm), a high degree of uniformity and a close match with the digital reference are also observed. The surface inside each square is uniform, with visually imperceptible deviations from the target value in the darkness. This varnish thickness does not cause print deformation. This further confirms the good compatibility of the black foil, the matte substrate, and this UV varnish layer. The measured dark values are *D*_S4_D_ = 100.0%, except at the edges of the printing elements.

The results of profiles show that all achieved varnish applications allow for highly precise transfer of larger graphic elements measuring 1 cm^2^ onto a matte-coated substrate. Smaller deviations are visible at the smallest application (7 μm) as minor internal surface instability. All generated profiles remain closely aligned with the reference, with no visible widening, narrowing, or edge deformation.

**Figure 10:**
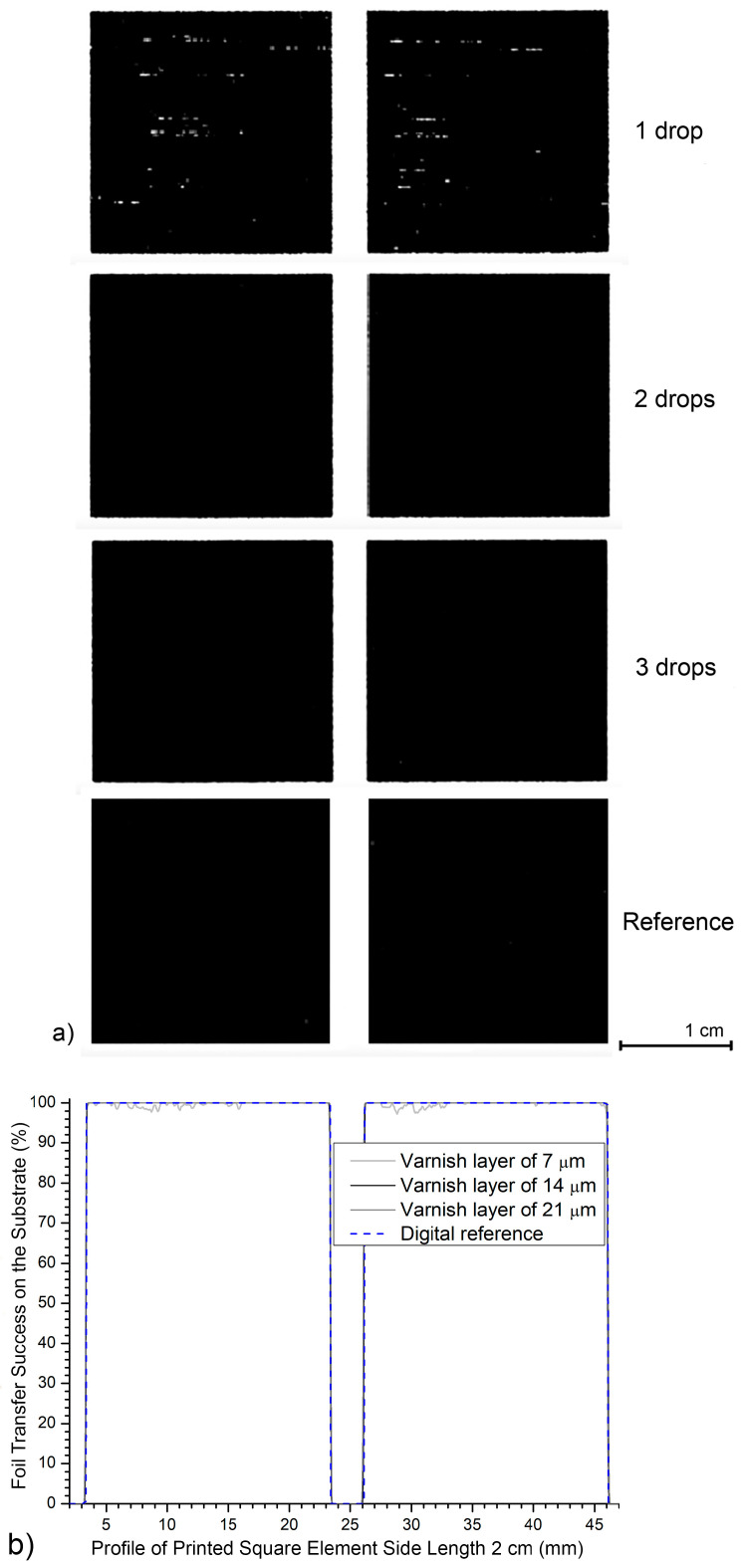
Profile of two printed elements created on a matte-coated substrate on a surface 5 cm long: (**a**) photo of a sample magnified 2×; (**b**) generated profiles.

Figure 10 shows a black imprint made by digital thermal transfer on matte paper. The analysis examines three UV varnish thicknesses (7, 14 and 21 µm) and compares these profiles with an ideal reference square. This representation helps assess the stability and uniformity of black prints on large surfaces.

At the smallest UV varnish thickness (7 μm), more pronounced internal micro deviations from the target value in the darkness value is observed on the surface printed with black foil. The outer edges remain precise and aligned with the reference. However, occasional peak inkjet drops occur on the square surface, resulting in a lowest darkness value of *D*_S2_E_ = 97.2%. This is below the ideal value of *D*_S2_E_ = 100.0% and indicates uneven varnish transfer on larger surfaces when the UV varnish layer is thinner.

For a 14 μm UV varnish coating, the surface inside the printed elements reaches and maintains a maximum darkness value of *D*_S3_E_ = 100.0% (without significant deviations from the target value). The edges of the black squares precisely follow the reference shape, and the entire print demonstrates high stability and a balanced printed surface.

The results exhibit behavior similar to that of the 21 μm varnish layer. Here, too, a perfectly filled print surface is achieved with a darkness value of *D*_S4_E_ = 100.0%. The edges of the black elements remain sharp and precisely defined.

The profiles in Figure 9 confirm that thicker varnish layers (14 and 21 μm) enable optimal foil transfer even on large areas of the printing elements, without loss of homogeneity or edge definition. In contrast, a thinner layer (7 μm) shows slight limitations (2.8%) in varnish coverage and uniformity on larger printing elements.

## 5. Conclusions

The largest deviations from the target value and deviations from the reference values were noticed for the smallest elements (square area of 0.01 and 0.06 cm^2^), especially with the smallest UV varnish application (7 μm). Compared to the digital reference, the largest deviation in the realization of printed black elements on the matte-coated printing substrate is Δ*D*_S2_C_ = 2.1%.

With a double or triple increase in the thickness of the UV varnish (14 and 21 μm), a more stable and uniform print is recorded. On a matte printing substrate, the deviation is Δ*D*_S4_C_ = 0.1%. Although the results with a 21 μm coating are technically optimum, a 14 μm coating has proven to be visually satisfactory, as it ensures very good print quality with reduced varnish consumption, which is important for industrial applications.

The image analysis showed that matte cardboard provides a more stable, uniform, and faithful reproduction of printed elements, with minimal deviations in profile and surface realization. It has an average reduction of 2.9% at 7 μm, and only 0.1% at 14 μm and 21 μm.

The obtained results provide valuable guidelines for optimizing industrial printing where high precision is important (security printing, transpromo printing, packaging printing, and decorative effects).

In future research on new-generation JETvarnish machines, the team will mandatorily analyze cross-direction printing (CD) and the settings of the inkjet heads to realize tactile printed elements. There is a special potential to test the foil’s behavior with significantly thicker UV varnish coatings (e.g., 42 μm or more), which may require multiple passes of the printing substrate through the inkjet machine. In addition, research and a further check of the readability of specific QR codes using reflective metallized foils that can be applied with this printing technology are planned. The substrate surface treatment (uncoated, matte-coated, or gloss-coated cardboard) affects the UV varnish’s adhesion and potential shedding. This will also be analyzed in future, as well as long-term durability testing.

## Figures and Tables

**Table 1:** Values of the coverage (A%) of experimental square print elements in an area of 24.01 cm^2^.

Thickness of UV Varnish	Element of 0.01 cm^2^ (A)	Element of 0.06 cm^2^ (B)	Element of 0.25 cm^2^ (C)	Element of 1 cm^2^ (D)	Element of 4 cm^2^ (E)	Element of 16 cm^2^ (F)
(S1) Digital reference	28.27%	30.35%	33.27%	38.78%	68.26%	67.66%
(S2) 7 µm layer_1 drop	27.74%	29.89%	35.61%	38.44%	68.12%	67.27%
(S3) 14 µm layer _2 drops	29.06%	31.86%	37.14%	39.43%	68.69%	67.80%
(S4) 21 µm layer _3 drops	29.24%	33.94%	40.62%	39.89%	69.04%	67.87%

## Data Availability

The original contributions presented in this study are included in the article. Further inquiries can be directed to the corresponding author.

## References

[1] Maryann M. Modern Marketing with QR Codes. https://www.pbahealth.com/elements/modern-marketing-with-qr-codes.

[2] QRCode.com History of QR Code. https://www.qrcode.com/en/history.

[3] Maryann M. QR Code Implementation with GS1 Standards. https://www.theconsumergoodsforum.com/video/webinar-qr-code-implementation-with-gs1-standards.

[4] Jelić I., Vrkić D. (2013). QR codes in library—Does anyone use them?. Proceedings of the 36th International Convention on Information and Communication Technology, Electronics and Microelectronics (MIPRO).

[5] QRCode.com QR Code Specification. https://www.qrcode.com/en/about/standards.html.

[6] Wahsheh H., Luccio F. (2020). Security and Privacy of QR Code Applications: A Comprehensive Study, General Guidelines and Solutions. Information.

[7] Liu T., Yan B., Pan J.S. (2019). Color Visual Secret Sharing for QR Code with Perfect Module Reconstruction. Appl. Sci..

[8] E. Commission (2023). Directive of the European Parliament and of the Council on Driving Licences, Amending Directive (EU) 2022/2561 of the European Parliament and of the Council, Regulation (EU) 2018/1724 of the European Parliament and of the Council and Repealing Directive 2.

[9] E. Commission (2024). Regulation (EU) 2025/40 of the European Parliament and of the Council on Packaging and Packaging Waste, Amending Regulation (EU) 2019/1020 and Directive (EU) 2019/904.

[10] (2024). Information Technology: Automatic Identification and Data Capture Techniques—QR Code Bar Code Symbology Specification.

[11] (2024). Information Technology: Automatic Identification and Data Capture Techniques—Bar Code Symbol Print Quality Test Specification—Two-Dimensional Symbols.

[12] (1999). Information Technology: 8-Bit Single-Byte Coded Graphic Character Sets—Part 5: Latin/Cyrillic Alphabet.

[13] (1991). Information Technology: ISO 7-Bit Coded Character Set for Information Interchange.

[14] (1992). Information Technology: Control Functions for Coded Character Sets.

[15] (1998). Information Technology: 8-Bit Dingle-Byte Coded Graphic Character Sets—Part 1 Latin/Cyrillic Alphabet.

[16] (2024). Information Technology: Automatic Identification and Data Capture Techniques—Data Matrix Bar Code Symbology Specification.

[17] QRCode.com Printer Head Density and Module Size. https://www.qrcode.com/en/howto/cell.html.

[18] (2024). International Symbology Specification.

[19] (2025). Information Technology: Automatic Identification and Data Capture Techniques—Data Carrier Identifiers (Including Symbology Identifiers).

[20] Kipphan H. (2001). Handbook of Print Media.

[21] Scodix Scodix Technology. https://scodix.com/printing-solutions/scodix-technology.

[22] MGI Company MGI: Digital Embellishment. https://mgi-fr.com/en/products/ennoblement-digital/.

[23] Duplo company DuSense DDC-810. https://duplointernational.com/article/everything-you-need-know-about-duplo-dusense-duplo.

[24] Majnarić I., Morić M., Valdec D., Milković K. (2024). The Effect of Applying UV LED-Cured Varnish to Metalized Printing Elements during Cold Foil Lamination. Coatings.

[25] QRCode.com Types of QR Code. https://www.qrcode.com/en/codes/.

[26] (1999). Information Technology: 8-Bit Single-Byte Coded Graphic Character Sets—Part 9: Latin Alphabet No. 5.

[27] Scodix Discover Scodix Cast & Cure Plus Other Leading Innovations in Digital Printing at PRINT 18. https://scodix.com/discover-scodix-castcure-plus-other-leading-innovations-in-digital-printing-at-print-18.

[28] Konica Minolta AIS SmartScaner. https://www.printingnews.com/software-workflow/product/12252965/mgi-digital-graphic-technology-ais-smartscanner.

[29] (2025). Information Technology: Automatic Identification and Data Capture Techniques—Direct Part Mark (DPM) Quality Guideline.

[30] (2025). Information Technology: Automatic Identification and Data Capture Techniques—Bar Code Print Quality Test Specification—Linear Symbols.

[31] (2007). Information Technology: Automatic Identification and Data Capture Techniques—Code 128 Bar Code Symbology Specification.

[32] Zebra Company DS2200 Series 1D/2D Scanners. http://www.zebra.com/content/dam/zebra_dam/en/spec-sheets/ds2200-series-spec-sheet-en-us.pdf.

[33] Honeywell Healthcare Scanners. https://automation.honeywell.com/us/en/products/productivity-solutions/barcode-scanners/healthcare-scanners.

[34] Datalogic Datalogic Products. https://www.datalogic.com/eng/products-hp-16.html.

[35] Cognex Advanced Machine Vision Made Easy. https://www.cognex.com.

[36] REA Company REA VeriCube. http://www.barcodesinc.com/media/pdf/REA-JET/vericube-1.pdf?srsltid=AfmBOoprIiLjm0CWZnvKEOwlzgfmTqy0rGXpRCjhm6qT_FFPKNrCQu4h.

[37] Havenko S., Ohirko M., Ryvak P., Kotmalova O. (2020). Determining the factors that affect the quality of test prints at flexographic printing. East.-Eur. J. Enterp. Technol..

[38] Tse M.K., Klein A.H. Automated Test Equipment for the Development of Media for Digital Printing. Proceedings of the ICPS 98: International Congress on Imaging Science.

[39] Grice J., Allebach J.P. (1999). The Print Quality Toolkit: An integrated print quality assessment tool. J. Imaging Sci. Technol..

[40] Kraushaar A. (2025). PSD Process Standard Digital.

[41] Kutschera E.L., Crowell T.L. (2024). Scanning & sustainability: The role of QR codes in environmental consciousness of apparel consumption. Environ. Dev. Sustain..

[42] Nofal R.M. (2020). Initiating android phone technology using QR codes to make innovative functional clothes. Int. J. Cloth. Sci. Technol..

[43] Özyazgan V., Uzun V., Bilgin S. (2016). Evaluation of the QR Code Fabric Tag System for Textile Companies in Turkey. Tekst ve Mühendis..

[44] Park S., Kim J. (2016). Study on the Recognition Rate of Printed QR Codes by Digital Transfer Textile Printing -Focused on Changes in the Fineness and Color of Filament. Textile. Fash Bus..

[45] MGI Company MGI: A Printers Guide, JETvarnish Series—Best Practices. https://konicaminolta.ca/wps/wcm/connect/bca/9b7f86ef-3b37-4436-9519-f5e6f2c5e474/MGIJETvarnish3DSeries_A_PRINTERS_GUIDE_V1.4_160720.pdf?MOD=AJPERES&CONVERT_TO=url&CACHEID=ROOTWORKSPACE.Z18_0IDCHAS0L03T10A5N5R0IT3PU6-9b7f86ef-3b37-4436-9519-f5e6f2c5e474-n.

[46] MGI Company MGI JETvarnish 3D: Digital Varnish & Foil. https://mgi-fr.com/wp-content/uploads/2022/02/MGI-Brochure-JETvarnish-3D-FR-SD.pdf.

[47] Konica Minolta Inkjet Print Head KM1024i Series. https://www.konicaminolta.com/global-en/inkjethead/products/inkjethead/km1024i/spec.html.

